# Three new species of *Cudonia (Rhytismatales)* from the Qinghai-Xizang Plateau, China

**DOI:** 10.3897/mycokeys.134.197873

**Published:** 2026-06-15

**Authors:** Jin-Rong Lu, Feng-Ming Yu, Jin-Ming Zhang, Guo-Lian Xu, Qi Zhao, Jun-Feng Liang

**Affiliations:** 1 Research Institute of Tropical Forestry, Chinese Academy of Forestry, Guangzhou 510520, China Institute of Vegetable Research, Gansu Academy of Agricultural Sciences Lanzhou China; 2 State Key Laboratory of Phytochemistry and Natural Medicines, Yunnan Key Laboratory for Fungal Diversity and Green Development, Kunming Institute of Botany, Chinese Academy of Sciences, Kunming 650201, China Research Institute of Tropical Forestry, Chinese Academy of Forestry Guangzhou China; 3 Institute of Vegetable Research, Gansu Academy of Agricultural Sciences, Lanzhou 730070, China State Key Laboratory of Phytochemistry and Natural Medicines, Yunnan Key Laboratory for Fungal Diversity and Green Development, Kunming Institute of Botany, Chinese Academy of Sciences Kunming China; 4 Department of Journal of Southwest Forestry University, Kunming 650233, China Department of Journal of Southwest Forestry University Kunming China

**Keywords:** Genetic distance, morphology, phylogenetic studies, species diversity, systematics

## Abstract

*Cudonia* is a small-sized member of the family *Cudoniaceae (Rhytismatales)*, comprising 19 recognized species that are known for producing small, club-shaped or rounded, faint yellow, buff-to-brown fruiting bodies commonly found in temperate regions of the Northern Hemisphere. Three new species of *Cudonia* were discovered in this study from the mid-montane and subalpine regions of the Qinghai-Xizang Plateau. These species were described based on morphological characteristics and molecular data. Two of the new species, *C. pallida* and *C. subalpina*, corresponded, respectively, to the previously documented phylogenetic species “*Cudonia* sp. 1” and “*Cudonia* sp. 3”. The third species, *C. longispora*, formed a distinct and well-supported lineage. The findings of this study indicated that the Qinghai-Xizang Plateau is a center of *Cudonia* species diversification and harbors rich floristic diversity, as has been proposed previously. It is hypothesized that novel species lineages still exist in this region.

## Introduction

*Cudonia* Fr. is a saprotrophic genus typified by *C. circinans* (Pers.) Fr., comprising 19 recognized species (Wang et al. 2002; Ge et al. 2014; Zheng et al. 2020; Yang 2023; Hyde et al. 2024; Burenbaatar et al. 2025; Niu et al. 2025; Lu et al. 2026; Shen et al. 2026). Morphologically, the genus is characterized by capitate, rounded, and club-shaped ascocarps with a well-developed stipe; clavate, 8-spored asci with non-bluing pores in Melzer’s reagent and sometimes filled with ascoconidia; filiform paraphyses, branched below, with the apical portion curved to circinate or straight above; and filiform or acicular, hyaline ascospores, some with gelatinous caps, gelatinous sheaths, or both (Zhuang 1998; Yang 2023). In certain species (e.g., *C. furfuracea* and *C. sichuanensis*), a stromatic membrane covers the hymenium in the early stage of ascoma development and is present between the ascigerous head and the stipe in some cases (Wang et al. 2002; Yang 2023). Ecologically, members of *Cudonia* are restricted to the Northern Hemisphere, showing the highest species diversity in temperate habitats, with only a few species reported from subtropical areas (Ge et al. 2014). They typically grow scattered to gregarious or in clusters in coniferous forests, often on humus layers with mosses and occasionally on well-rotted wood (Zhuang 1998; Ge et al. 2014).

In the pre-molecular era, the taxonomic position of *Cudonia* was unclear and long debated. Traditionally, the genus was arranged in the family *Geoglossaceae* or in *Leotiaceae* based on morphological characteristics such as pileate ascocarps, ascospore color, and the reaction of the ascus pore in Melzer’s reagent (Durand 1908; Corner 1929, 1930; Mains 1940, 1955; Imai 1941; Maas Geesteranus 1964; Korf 1973). Lizon et al. (1998) removed *Cudonia* from *Leotiaceae*, restricting the family to a narrow sense characterized by a well-developed gelatinized layer of textura intricata in the ectal excipulum. Nannfeldt (1942) suggested that *Cudonia* and *Spathularia* Pers. share key features with members of *Phacidiaceae* (including *Rhytisma* Fr., now a member of *Rhytismataceae*, *Rhytismatales*), such as filiform, branched, and circinate paraphyses, as well as a stromatic layer. This hypothesized relationship was later supported by molecular phylogenetic evidence (Gernandt et al. 2001; Wang et al. 2002; Ge et al. 2014). The family *Cudoniaceae (Rhytismatales)* was erected by Cannon to accommodate the genera *Cudonia* and *Spathularia* (Kirk et al. 2001). However, subsequent phylogenetic studies have challenged the circumscription of these genera. Based on multigene molecular phylogenetic analyses (ITS, LSU, *rpb*2, and *tef*-1α), the genus *Cudonia* and the family *Cudoniaceae* were supported as monophyletic groups, whereas the genus *Spathularia* was not (Ge et al. 2014). Given the few diagnostic morphological characters available to reliably distinguish *Cudonia* from *Spathularia*, Ge et al. (2014) suggested merging the two genera, with the name *Spathularia* taking nomenclatural priority. The micromorphology of these two genera is similar, featuring curved paraphyses, club-shaped asci, and long, thin, hyaline ascospores that are enveloped in gelatinous sheaths (Ge et al. 2014). In contrast, *Cudonia* differs from *Spathularia* in the morphology of their ascomata, as *Spathularia* species have flattened fruiting bodies (e.g., spatula-like), whereas *Cudonia* species bear rounded and club-shaped fruiting bodies, although some *Cudonia* species are morphologically unique and not easily pigeonholed into either genus (e.g., *C. sichuanensis* and *C. claviformis*) (Wang et al. 2002; Ge et al. 2014; Yang 2023).

Using a combined dataset of ITS, LSU, *rpb*2, and *tef*-1α sequences obtained from 111 collections across the Northern Hemisphere, Ge et al. (2014) constructed a phylogeny of *Cudonia* and *Spathularia* and circumscribed 32 species-level clades, including 23 putative undescribed phylogenetic species. Yang (2023) made the nomenclatural treatment for two phylogenetic species within *Cudonia*, formally naming them as *C. claviformis* and *C. furfuracea*. Subsequently, six other new species were proposed in some studies (Hyde et al. 2024; Burenbaatar et al. 2025; Niu et al. 2025; Lu et al. 2026; Shen et al. 2026). In China, 13 species have been reported, with high species-level diversity in southwestern regions. During the study of macrofungi in southwestern China, three undescribed species of *Cudonia* were uncovered from Gansu, Xizang, and Yunnan. They are described and illustrated herein.

## Materials and methods

### Sample collection and morphological studies

Eight specimens of *Cudonia* were collected, one of which (HKAS 154397) was from Gansu, two (HKAS 146472 and HKAS 146473) were from Yunnan, and the rest (HKAS 135870, HKAS 135871, HKAS 135872, HKAS 135873, and HKAS 135874) were from Xizang, China. The specimens were photographed in situ and dried using a mushroom dryer. The macromorphological characters were described based on fresh and dried samples. The color of the ascomata was determined following Kornerup and Wanscher (1967). Microscopic structures were observed with light microscopy from dried specimens after hand sectioning and mounting in distilled water. Melzer’s reagent was used to test the amyloid nature of the asci. A charge-coupled device SC 2000C attached to a Nikon ECLIPSE Ni-U compound microscope (Model Eclipse Ni-U, Nikon Corporation, Tokyo, Japan) was used to examine the characters of the hymenium, excipulum, asci, paraphyses, ascospores, and ascoconidia. The notations “asci, ascospores, and ascoconidia (*n*/*m*/*p*)” indicate that the measurements of each structure were made on *n* units (asci, ascospores, or ascoconidia) derived from *m* ascocarps of *p* collections. All measurements were recorded using the Tarosoft (R) Image Framework program (IFW), and images were processed with Adobe Photoshop 2019 (Adobe Systems, USA). Dried specimens were deposited at the Herbarium of Cryptogams, Kunming Institute of Botany, Academia Sinica (KUN-HKAS).

### DNA extraction, PCR amplification, and sequencing

Genomic DNA was extracted from dried fruiting bodies using the TriliefTM Plant Genomic DNA Kit (Tsingke Biological Technology Co., Ltd., Beijing, China). The primer pairs ITS1-F/ITS4 (White et al. 1990; Gardes and Bruns 1993), LR0R/LR5 (Vilgalys and Hester 1990), EF1-983F/EF1-2218R (Rehner and Buckley 2005), and fRPB2-5F/fRPB2-7R (Liu et al. 1999) were used for the amplification of the internal transcribed spacer region of ribosomal DNA (ITS1-5.8S-ITS2, ITS), the partial nuclear large subunit rDNA gene region (LSU), the translation elongation factor-1α gene (*tef*-1α), and the RNA polymerase II second-largest subunit (*rpb*2), respectively. Polymerase chain reaction (PCR) was performed in a total volume of 25 μL, containing 21 μL of 1.1 × T3 Super PCR Mix (Tsingke TSE030, Tsingke Biological Technology Co.), 1 μL of each primer, and 2 μL of DNA template. PCR reactions were carried out in an Applied Biosystems 2720 thermocycler (Foster City, CA, USA) under the following conditions: initial denaturation at 98 °C for 5 min, followed by 35 cycles of denaturation at 98 °C for 20 s, annealing at 53 °C for 15 s and extension at 72 °C for 25 s, followed by a final extension at 72 °C for 7 min for ITS and LSU. The PCR reaction conditions for *tef*-1α and *rpb*2 were as follows: 5 min at 98 °C, followed by 35 cycles of 40 s at 98 °C, 30 s at 55 °C and 40 s at 72 °C, and a final extension of 7 min at 72 °C. PCR products were verified by electrophoresis with a 1% ethidium bromide-stained agarose gel (Innis et al. 2012). Successful PCR products were sent to Sangon Biotech (Shanghai) Co., Ltd., Shanghai, China, for sequencing.

### Sequence assembly, alignment, and phylogenetic analyses

The phylogenetic trees were constructed using the sequencing data of newly collected samples and their allied reference sequences downloaded from GenBank (Table 1). *Spathularia velutipes* (H337, H338, H339, S3, and S4) was used as the outgroup taxon. All sequences were assembled and aligned using MAFFT v. 7 (Kuraku et al. 2013; Katoh et al. 2019) and manually edited when necessary in BioEdit version 7.0.9 (Hall 1999). Individual alignments were compiled for ITS, LSU, *rpb*2, and *tef*-1α loci. The optimal substitution model for each dataset was determined using MrModeltest 2.3 (Nylander 2004) under the Akaike information criterion (AIC). The results indicated that SYM+I+G was optimal for the ITS and *tef*-1α partitions, and HKY+I+G for the LSU and *rpb*2 partitions. The individual datasets were then concatenated in the order ITS + LSU + *rpb*2 + *tef*-1α to assemble the combined dataset.

**Table 1.** Species names, sample ID/voucher, and corresponding GenBank accession numbers of the taxa used in this study. The letters ^HT^ after the sample indicate the holotype. Specimens of the current study are in bold.

Species name	Herbarium/collection no. (typification)	Geographical origin	GenBank accession no.				Reference
			ITS	LSU	*rpb*2	*tef*-1α	
*Cudonia aurantiaca*	T587 ^HT^	China: Zhejiang	PZ070749	PZ125084	PZ124377	PZ124383	Shen et al. (2026)
*C. aurantiaca*	T5872	China: Zhejiang	PZ070750	/	PZ124378	PZ124384	Shen et al. (2026)
*C. cangshanensis*	KUN-HKAS 147014 ^HT^	China: Yunnan	PV607235	/	/	/	Niu et al. (2025)
*C. cangshanensis*	KUN-HKAS 147015	China: Yunnan	PV607236	/	/	/	Niu et al. (2025)
*C. circinans*	C316	Switzerland	KC833156	KC833182	KC833275	KC833349	Ge et al. (2014)
*C. circinans*	C317	Switzerland	KC833157	KC833183	KC833276	KC833350	Ge et al. (2014)
*C. circinans*	C319	Switzerland	KC833158	KC833184	KC833277	KC833351	Ge et al. (2014)
*C. claviformis*	KUN-HKAS 45676 (H346) ^HT^	China: Xizang	KC833169	KC833213	KC833298	KC833380	Ge et al. (2014); Yang (2023)
*C. confusa*	C314	Finland	KC833165	KC833216	KC833300	KC833383	Ge et al. (2014)
*C. confusa*	C315	Finland	KC833166	KC833217	/	KC833384	Ge et al. (2014)
*C. confusa*	C318	Switzerland	KC833167	/	/	/	Ge et al. (2014)
*C. furfuracea*	KUN-HKAS 45675 (H348) ^HT^	China: Xizang	KC833136	KC833203	KC833296	KC833369	Ge et al. (2014); Yang (2023)
*C. furfuracea*	KUN-HKAS 49324 (C287)	China: Sichuan	KC833134	KC833201	KC833294	KC833368	Ge et al. (2014); Yang (2023)
*C. furfuracea*	KUN-HKAS 46020 (C288)	China: Xizang	KC833135	KC833202	KC833295	KC833370	Ge et al. (2014); Yang (2023)
*C. gracilistipitata*	KUN-HKAS 129661 ^HT^	China: Yunnan	OR500915	/	/	/	Hyde et al. (2024)
*C. gracilistipitata*	KUN-HKAS 129661	China: Yunnan	OR500916	/	/	/	Hyde et al. (2024)
*C. linzhiensis*	KUN-HKAS 133627 ^HT^	China: Xizang	PP853392	PP851389	/	/	Lu et al. (2026)
*C. linzhiensis*	KUN-HKAS 133645	China: Xizang	PP853393	PP851390	/	/	Lu et al. (2026)
***C. longispora***	**KUN-HKAS 146472 ^HT^**	**China: Yunnan**	**PV364313**	**PV362122**	**PZ482076**	**PZ482068**	**This study**
***C. longispora***	**KUN-HKAS 146473**	**China: Yunnan**	**PV364314**	**PV362123**	**PZ482077**	**PZ482069**	**This study**
*C. lutea*	C280	China: Sichuan	KC833150	KC833186	/	/	Ge et al. (2014)
*C. lutea*	C283	China: Sichuan	KC833151	KC833187	/	/	Ge et al. (2014)
*C. mongolica*	HMJAU 71948 ^HT^	Mongolia	PQ045731	PQ045733	PQ057764	PQ057769	Burenbaatar et al. (2025)
*C. mongolica*	2380202	Mongolia	PQ045732	PQ045734	PQ057765	PQ057770	Burenbaatar et al. (2025)
***C. pallida***	**KUN-HKAS 135873 ^HT^**	**China: Xizang**	**PZ099287**	**PZ092773**	**PZ482078**	**PZ482070**	**This study**
***C. pallida***	**KUN-HKAS 135874**	**China: Xizang**	**PZ099288**	**PZ092774**	**PZ482079**	**PZ482071**	**This study**
***C. pallida***	**KUN-HKAS 154397**	**China: Gansu**	**PZ342139**	**PZ336248**	**PZ482080**	**PZ482072**	**This study**
*C. sichuanensis*	C292	China: Yunnan	KC833121	KC833218	KC833301	KC833385	Ge et al. (2014)
*C. sichuanensis*	C331	China: Yunnan	KC833123	KC833219	/	/	Ge et al. (2014)
*C. sichuanensis*	C328	China: Yunnan	KC833122	KC833220	KC833302	KC833386	Ge et al. (2014)
*C. sichuanensis*	S66	China: Sichuan	KC833120	/	/	/	Ge et al. (2014)
***C. subalpina***	**KUN-HKAS 135870**	**China: Xizang**	**PZ099284**	**PZ092770**	**PZ482073**	**PZ482065**	**This study**
***C. subalpina***	**KUN-HKAS 135871 ^HT^**	**China: Xizang**	**PZ099285**	**PZ092771**	**PZ482074**	**PZ482066**	**This study**
***C. subalpina***	**KUN-HKAS 135872**	**China: Xizang**	**PZ099286**	**PZ092772**	**PZ482075**	**PZ482067**	**This study**
*C. yunnanensis*	KUN-HKAS 129663 ^HT^	China: Yunnan	OR500917	/	/	/	Hyde et al. (2024)
*C. yunnanensis*	KUN-HKAS 129664	China: Yunnan	OR500918	/	/	/	Hyde et al. (2024)
*Cudonia* sp. 1	C281	China: Sichuan	KC833146	KC833191	KC833284	KC833358	Ge et al. (2014)
*Cudonia* sp. 1	H672	China: Yunnan	KC833145	KC833192	KC833285	KC833359	Ge et al. (2014)
*Cudonia* sp. 1	H664	China: Yunnan	KC833144	KC833193	KC833286	KC833360	Ge et al. (2014)
*Cudonia* sp. 1	C312	China: Yunnan	KC833143	KC833194	KC833291	KC833361	Ge et al. (2014)
*Cudonia* sp. 1	C286	China: Xizang	KC833139	KC833195	KC833287	KC833362	Ge et al. (2014)
*Cudonia* sp. 1	C289	China: Sichuan	KC833140	KC833196	KC833288	KC833363	Ge et al. (2014)
*Cudonia* sp. 1	C290	China: Sichuan	KC833141	KC833197	KC833289	KC833364	Ge et al. (2014)
*Cudonia* sp. 1	C291	China: Xizang	KC833142	KC833198	KC833290	KC833365	Ge et al. (2014)
*Cudonia* sp. 2	C282	China: Sichuan	KC833148	KC833188	KC833281	KC833355	Ge et al. (2014)
*Cudonia* sp. 2	H675	China: Yunnan	KC833149	KC833189	KC833282	KC833356	Ge et al. (2014)
*Cudonia* sp. 3	C306	China: Jilin	KC833163	KC833175	KC833269	KC833343	Ge et al. (2014)
*Cudonia* sp. 3	H674	China: Yunnan	KC833164	KC833176	/	KC833344	Ge et al. (2014)
*Cudonia* sp. 3	H666	China: Yunnan	KC833162	KC833177	KC833270	KC833345	Ge et al. (2014)
*Cudonia* sp. 3	H660	China: Yunnan	KC833161	KC833178	KC833271	KC833346	Ge et al. (2014)
*Cudonia* sp. 3	H654	China: Yunnan	KC833160	KC833179	KC833272	KC833347	Ge et al. (2014)
*Cudonia* sp. 3	C325	China: Yunnan	KC833159	KC833180	KC833273	/	Ge et al. (2014)
*Cudonia* sp. 3	S11	China: Jilin	KC833173	KC833268	/	KC833434	Ge et al. (2014)
*Cudonia* sp. 4	C285	China: Xizang	KC833127	KC833209	KC833324	KC833376	Ge et al. (2014)
*Cudonia* sp. 4	H605	China: Gansu	KC833128	KC833210	KC833322	KC833377	Ge et al. (2014)
*Cudonia* sp. 5	H658	China: Yunnan	KC833147	KC833190	KC833283	KC833357	Ge et al. (2014)
*Cudonia* sp. 6	1222	China: Yunnan	KC833138	KC833200	KC833293	KC833367	Ge et al. (2014)
*Cudonia* sp. 6	H680	China: Yunnan	KC833137	KC833199	KC833292	KC833366	Ge et al. (2014)
*Cudonia* sp. 7	H670	China: Jilin	KC833152	KC833185	KC833278	KC833352	Ge et al. (2014)
*Cudonia* sp. 8	H342	USA	KC833133	KC833204	KC833297	KC833371	Ge et al. (2014)
*Cudonia* sp. 9	H371	China: Neimengu	KC833124	KC833214	KC833299	KC833381	Ge et al. (2014)
*Cudonia* sp. 11	H027	Canada	KC833153	/	/	/	Ge et al. (2014)
*Cudonia* sp. 11	H340	USA	KC833155	KC833181	KC833274	KC833348	Ge et al. (2014)
*Cudonia* sp. 11	H037	USA	KC833154	/	/	/	Ge et al. (2014)
*Cudonia* sp. 11	S9	USA	KC833174	/	/	KC833435	Ge et al. (2014)
*Cudonia* sp. 13	H357	China: Xizang	KC833131	KC833205	KC833325	KC833374	Ge et al. (2014)
*Cudonia* sp. 13	H361	China: Xizang	KC833132	KC833206	/	KC833372	Ge et al. (2014)
*Cudonia* sp. 13	H362	China: Xizang	KC833130	KC833207	KC833321	KC833373	Ge et al. (2014)
*Cudonia* sp. 13	C284	China: Xizang	KC833129	KC833208	KC833323	KC833375	Ge et al. (2014)
*Cudonia* sp. 14	H360	China: Sichuan	KC833126	KC833211	/	KC833378	Ge et al. (2014)
*Cudonia* sp. 15	C304	China: Xizang	KC833125	KC833212	KC833320	KC833379	Ge et al. (2014)
*Spathularia velutipes*	S3	USA	KC833171	KC833265	KC833328	KC833409	Ge et al. (2014)
*S. velutipes*	S4	USA	KC833172	KC833266	/	KC833431	Ge et al. (2014)
*S. velutipes*	H337	USA	KC833088	KC833239	/	KC833432	Ge et al. (2014)
*S. velutipes*	H338	USA	KC833089	KC833240	KC833326	KC833407	Ge et al. (2014)
*S. velutipes*	H339	USA	KC833086	KC833241	KC833327	KC833408	Ge et al. (2014)

Maximum likelihood (ML) analysis was performed using IQ-Tree (http://iqtree.cibiv.univie.ac.at/, accessed on 23 May 2026) (Trifinopoulos et al. 2016). The substitution model options for each locus were automatically evaluated according to the provided partition file. Clade support for the ML analysis was assessed using an SH-aLRT test with 1,000 replicates (Guindon et al. 2010) and ultrafast bootstrap (UFB) (Hoang et al. 2018). In the ML analysis, nodes with support values of both SH-aLRT ≥ 80 and UFB ≥ 95 were well-supported, those with either SH-aLRT < 80 or UFB < 95 were weakly supported, and nodes with both SH-aLRT < 80 and UFB < 95 were unsupported.

Bayesian inference (BI) posterior probability (Rannala and Yang 1996) was performed with Markov chain Monte Carlo sampling (MCMC) to evaluate posterior probability using MrBayes version 3.2.7 (Ronquist et al. 2012). Four simultaneous MCMC chains were run for 30,000,000 generations and sampled every 1,000 generations until the standard deviation of split frequencies fell below 0.01 and ESS values were > 200 (Ronquist et al. 2012). Subsequently, phylogenetic trees were summarized and posterior probabilities (PP) were calculated using MCMC by discarding the first 25% of sampled generations as the burn-in phase. Nodes were considered strongly supported with posterior probability values greater than 0.95. The phylogenetic tree was visualized in FigTree v.1.4.4 (Rambaut 2018).

## Results

### Phylogenetic analyses

**Figure 1.**
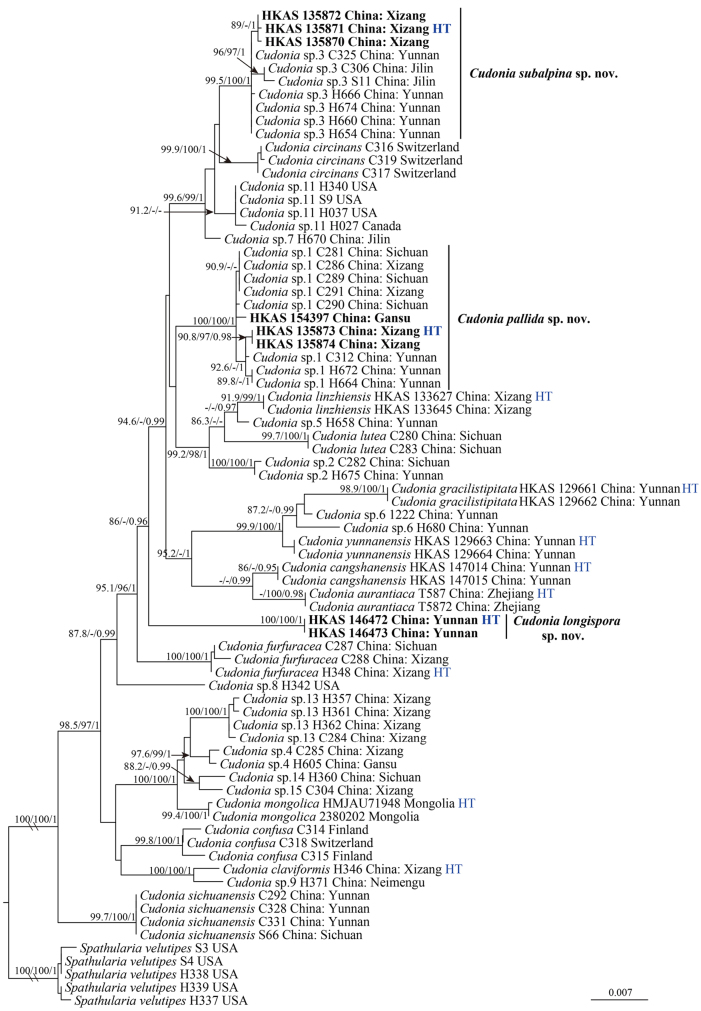
Maximum likelihood (ML) tree inferred from a combined ITS, LSU, *rpb*2, and *tef*-1α sequence dataset of representative specimens of *Cudonia* species. *Spathularia velutipes* (H337, H338, H339, S3, and S4) was used as the outgroup taxon. Bootstrap support values for ML ≥ 80 of SH-aLRT or 95 of UFB and posterior probability for BIPP ≥ 0.95 are indicated above the nodes and separated by “-/-/-” (SH-aLRT/UFB/BIPP). Specimens of the current study are in bold. The letter HT after the sample indicates the holotype specimen.

Phylogenetic analyses were based on a combined dataset of ITS + LSU + *rpb*2 + *tef*-1α dataset from 76 taxa across the family *Cudoniaceae*, including eight newly collected specimens in the present study. Five specimens of *Spathularia velutipes* were selected as the outgroup. The combined alignment comprised 2,567 characters with gaps (ITS 531 bp, LSU 859 bp, *rpb*2 694 bp, *tef*-1α 483 bp). The dataset comprised 2,214 constant characters, 296 variable parsimony-informative characters, and 57 singleton characters. The maximum likelihood (ML) phylogenetic tree is presented in Fig. 1.

Phylogenetic analyses revealed that the eight newly collected specimens fell into three distinct lineages, representing two previously documented phylogenetic species and three undescribed species. The first undescribed species was represented by three specimens (HKAS 135870, HKAS 135871, HKAS 135872), which clustered within the lineage of the phylogenetic species *Cudonia* sp. 3 with strong statistical support (SH-aLRT = 99.5, UFB = 100, BIPP = 1). This species, *C. subalpina*, together with *C. circinans* and two other phylogenetic species (*Cudonia* sp. 11 and *Cudonia* sp. 7), formed a robust branch with high support values (SH-aLRT = 99.6, UFB = 99, BIPP = 1). Their topology is the same as that reported in Ge et al. (2014). A second lineage corresponded to *Cudonia* sp. 1, where three specimens (HKAS 135873, HKAS 135874, HKAS 154397) were placed with high support (SH-aLRT = 100, UFB = 100, BIPP = 1). This lineage was formally described here as *C. pallida*. It was sister to a clade comprising the recently documented species *C. linzhiensis*, the old species *C. lutea*, and two phylogenetic species (*Cudonia* sp. 5 and *Cudonia* sp. 2), although this relationship lacked statistical support. The remaining two specimens (HKAS 146472, HKAS 146473) represented a third undescribed species, named here as *C. longispora*, which formed a strongly supported independent branch (SH-aLRT = 100, UFB = 100, BIPP = 1).

### Taxonomy

#### Cudonia longispora

Taxon classificationFungiRhytismatalesCudoniaceae

J.R. Lu, F.M. Yu, J.F. Liang & Q. Zhao sp. nov.

777FA9B7-FC0A-51BA-AD2E-835B8EAC8D79

Index Fungorum: IF905590

Fig. 2

##### Chinese name.

长孢地锤菌 (chang bao di chui jun).

**Figure 2.**
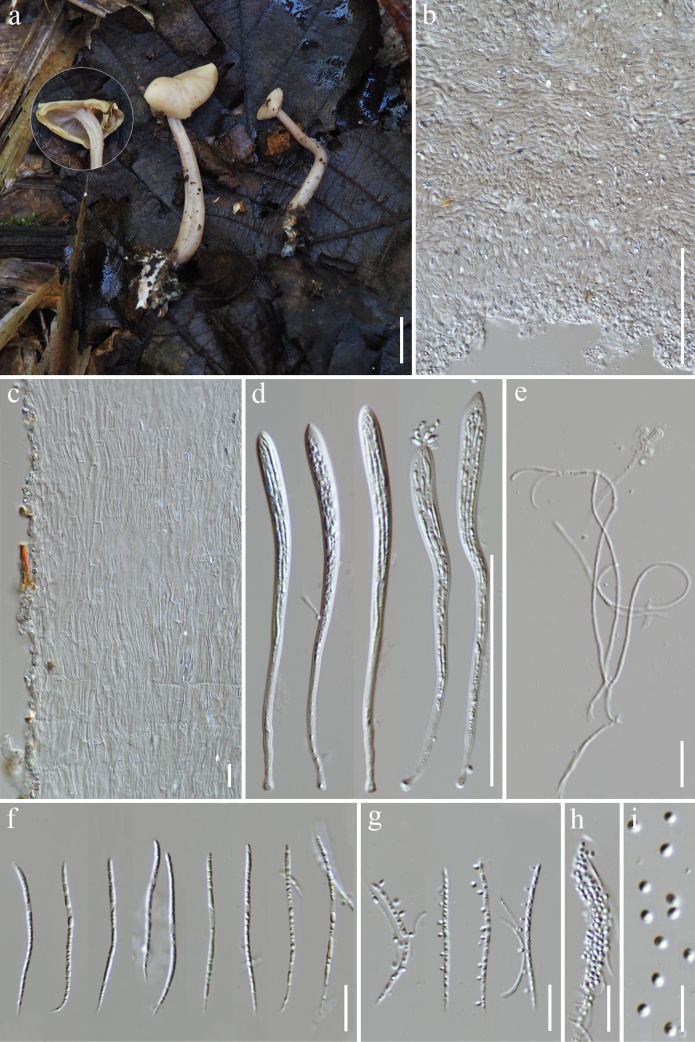
*Cudonia longispora* (HKAS 146472, holotype). **a**. Fresh fruiting bodies; **b**. Interior and outermost layer of the ascigerous portion; **c**. Longitudinal section of stipe showing the trama and surface (left side); **d**. Asci; **e**. Paraphyses; **f, g**. Ascospores; **h**. Ascoconidia in asci; **i**. Ascoconidia; Scale bars: 1 cm (**a**); 100 μm (**b, d**); 20 μm (**c, e–h**); 10 μm (**i**).

##### Etymology.

The specific epithet longispora (Latin, meaning “long” and “spore”) refers to this species bearing longer ascospores (58.5–73 µm in length).

##### Holotype.

China • Yunnan, Tengchong City, alt. 1,819 m, in a mixed forest with *Pinus yunnanensis*, *Quercus* trees, and bamboos, 12 November 2024, Jin-Rong Lu, LJR 907a (HKAS 146472).

##### Diagnosis.

Characterized by long ascospores (58.5–73 µm in length), each possessing multiple bud points for the production of ascoconidia, typically six or eight bud points per ascospore, whereas most other species have approximately four, except for *C. aurantiaca*, which has been reported to have over 30 bud points on a single ascospore.

##### Description.

***Ascocarp*** scattered, capitate, stipitate, 3.5–4.7 cm in height. ***Ascigerous portion*** thin, 1–2 cm in diam., sub-capitate, with margins folded downwards, triangular or saddle-shaped, slightly compressed; hymenium surface greyish green and greyish yellow when fresh, smooth, glabrous. ***Stipe*** 3–4 cm in height, 2.5–5 mm in diam., subcylindrical to cylindrical, slightly striate to ridged, solid but partially hollow in mature to over-mature specimens, erect or slightly bent, pale grey, grey, or yellow grey, smooth or slightly tomentose, especially on the upper part with whitish to brownish furfuraceous squamules. ***Stromatic membrane*** over the hymenium not seen.

***Hymenium*** 132–157 µm thick, consisting of paraphyses and asci at different stages of development. ***Asci*** [50/2/1] 100–184.5 × 7–11.5 µm, clavate, tapered towards the base, 8-spored, apical portion narrowly rounded, apical pore J^-^, croziers present. ***Ascospores*** [50/2/1] 58.5–73 × 2–3.5 µm, long-linear, filiform, often flexuous, hyaline and colorless, rounded at apex and acuminate at base, multi-guttulate, non-septate but becoming septate when forming ascoconidia. ***Ascoconidia*** [50/2/1] 3–4 × 2.5–3 µm, subglobose, limoniform, smooth, 1-celled, containing a large oil drop, colorless and hyaline; sometimes nearly filling the asci. ***Paraphyses*** filiform, uncommonly septate, branched mostly in the lower part, and occasionally with short binary branches in the upper part, curved in the upper part with the apical portion slightly swollen; middle portion 1.5–2.5 µm in diam., apical portion reaching up to 2.5 µm at the widest point, hyaline and colorless. ***Interior of ascigerous portion*** of *textura intricata* to *porrecta*, hyphae 3–6.5 µm in diam., arranged in parallel, relatively compressed, hyaline and colorless. ***Outermost layer of ascigerous portion*** (the side opposite the hymenium) composed of ellipsoid to rounded-angular cells, some elongated and hyphoid; hyphae 5.5–12 µm in diam., elongated cells 18–28 × 5.5–9 µm, elliptical to subglobular cells 6–14 × 4.5–8 µm, covered with a thin layer of small-sized ellipsoid to rounded-angular cells (2.5–8 × 2–4.5 µm) mixed with short, twisted hyphae (1.5–3 µm in diam.), hyaline and colorless. ***Interior of stipe*** of *textura porrecta*, with longitudinally and compactly arranged, septate hyphae 3–5 µm in diam., colorless, and hyaline; hyphae gradually widening toward the outside, 4–8 µm in diam., covered with a brown crust.

##### Ecology.

On soil covered with a thick layer of humus, scattered in subtropical coniferous and broad-leaf forests; fall.

##### Distribution.

China (western Yunnan), mid-montane, subtropical.

##### Additional collections examined.

China • Yunnan, Tengchong City, alt. 1,819 m, in a mixed forest with *Pinus yunnanensis*, *Quercus* trees, and bamboos, 12 November 2024, Jin-Rong Lu, LJR 907b (HKAS 146473).

##### Notes.

In phylogenetic analyses, specimens of *C. longispora* (HKAS 146472 and HKAS 146473) formed an independent lineage within *Cudonia* (Fig. 1). Morphologically, *C. longispora* resembles *C. japonica* in having a thin, saddle-shaped, triangular or irregularly convex, yellowish ascigerous portion and a subconcolorous stipe that is longitudinally furrowed above (Imai 1936). The latter species, *C. japonica*, is known only from Japan, and its molecular information is currently unavailable. The two species overlap in ascospore length (58.5–73 × 2–3.5 µm in *C. longispora* vs. 65–85 × 2.5–3.5 µm in *C. japonica*; Imai 1936). However, compared to *C. japonica*, *C. longispora* has slightly narrower asci [*C. longispora*: 7–11.5 µm wide; *C. japonica*: 12–14 µm wide in Yasuda (1915) and 10–15 µm wide in Imai (1936)]. The original description of *C. japonica* did not mention ascoconidia, making it impossible to compare the size of ascoconidia or the number of bud points that produce ascoconidia on its ascospores (Yasuda 1915; Imai 1936).

The number of bud points producing ascoconidia on ascospores may be a useful character for species identification in *Cudonia*. *Cudonia aurantiaca* was recently formally described from southeastern China (Shen et al. 2026). More than 30 bud points can be observed on a single ascospore of this species, as illustrated in Shen et al. (2026). *Cudonia longispora* and *C. aurantiaca* were not closely related in the phylogenetic tree. Compared to *C. longispora*, *C. aurantiaca* has typically light orange to golden yellow fruiting bodies and much smaller ascoconidia (1.1–2.4 × 1.1–2.1 µm; Shen et al. 2026).

#### Cudonia pallida

Taxon classificationFungiRhytismatalesCudoniaceae

J.R. Lu, F.M. Yu, J.F. Liang & Q. Zhao sp. nov.

AD46A548-B234-53AD-A5B3-CE848D845C91

Index Fungorum: IF905589

Fig. 3

##### Chinese name.

淡色地锤菌 (dan se di chui jun).

**Figure 3.**
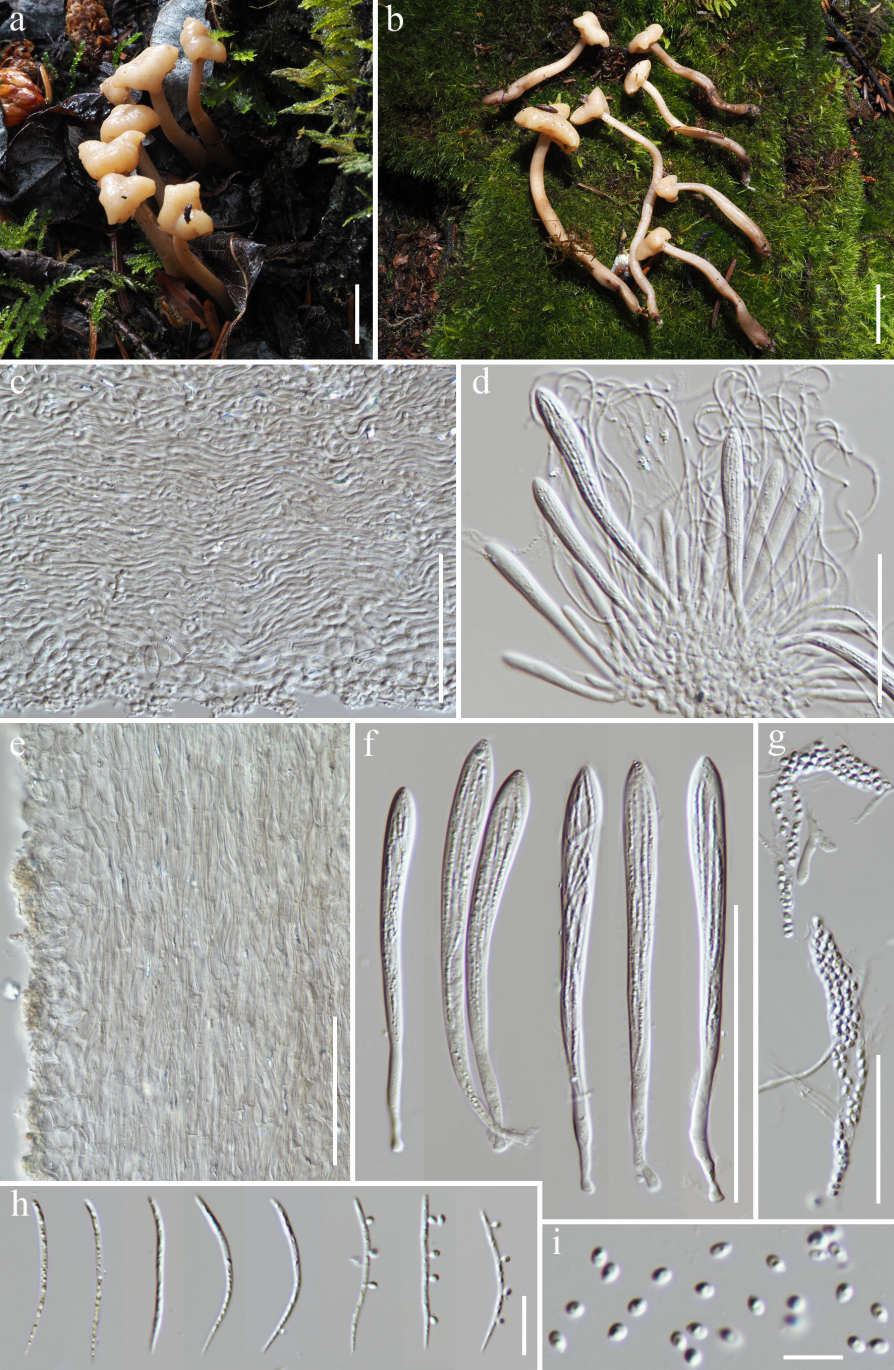
*Cudonia pallida* (HKAS 135873, holotype). **a, b**. Fresh fruiting bodies; **c**. Interior and outermost layer of the ascigerous portion; **d**. Asci and paraphyses; **e**. Longitudinal section of stipe showing the trama and surface (left side); **f**. Asci; **g**. Ascoconidia in asci; **h**. Ascospores, the right three ascospores producing ascoconidia; **i**. Ascoconidia. Scale bars: 1.5 cm (**a, b**); 100 μm (**c, e, f**); 50 μm (**d, g**); 20 μm (**h**); 10 μm (**i**).

##### Etymology.

The specific epithet pallida (Latin, meaning “pallid”) alludes to the pale yellow, yellowish white to orange white fruiting bodies.

##### Holotype.

China • Xizang, Linzhi City, Gongbujiangda County, alt. 3,498 m, in a mixed forest dominated by *Picea asperata*, with gravel trees and rosaceous shrubs, on soil surrounded by mosses and humus, 9 August 2025, Jin-Rong Lu, LJR 1573a (HKAS 135873).

##### Diagnosis.

Similar to *Cudonia fecunda* in gross features but having different anatomical structures of ascigerous portion and shorter ascospores.

##### Description.

***Ascocarp*** scattered to gregarious, capitate, stipitate, 3.5–6.5 cm in height. ***Ascigerous portion*** 1–2 cm in diam., subcapitate, margins folded downwards, triangular or saddle-shaped, hymenium surface pale yellow, yellowish white, to orange white when fresh, smooth. ***Stipe*** 3–6 cm in height, 2–4 mm in diam., subcylindrical to cylindrical, solid, erect or slightly bent, striate especially on the upper part, smooth or slightly tomentose, concolorous with the hymenium surface, to light brown, brown to dark brown. ***Stromatic membrane*** over the hymenium not observed.

***Hymenium*** 106–154.5 µm thick, consisting of paraphyses and asci at different stages of development. ***Asci*** [50/2/1] 91–144.5 × 8–12.5 µm, clavate, tapered towards the base, 8-spored, apical portion narrowly rounded, apical pore J^-^, croziers present. ***Ascospores*** [50/2/1] 44.5–60 × 2–3 µm, linear, clavate-filiform, acicular, often flexuous, hyaline and colorless, rounded at apex and acuminate at base, multi-guttulate, non-septate or septate. ***Ascoconidia*** [50/2/1] 3.5–4.5 × 2.5–3.5 µm, subglobose, obovoid, limoniform, smooth, 1-celled, containing a large oil drop, colorless and hyaline; sometimes nearly filling the asci. ***Paraphyses*** filiform, septate, branched in the lower part, upper part curved or moderately contorted; apical portion slightly or suddenly swollen or swollen repeatedly to form a moniliform (bead-like) chain, clavate, capitate to spherical; middle portion 1.5–2.5 µm diam., apical portion 2–3.5 µm diam. at the widest point, hyaline and colorless. ***Ascigerous portion*** approximately composed of three layers: interior layer consisted of a loosely interwoven *textura intricata*, hyphae 3–7.5 µm diam., colorless and hyaline, becoming gradually more compact and parallel to the surface toward the outside; medullar layer thinner, composed of gelatinized, ellipsoid to rounded-angular cells, some elongated and hyphoid; hyphae 4.5–9 µm in diam.; outermost layer discontinuous, gelatinized, composed of rounded-angular cells with 3–6.5 µm in diam., and short, twisted hyphae, hyaline and colorless. ***Interior of stipe*** of *textura intricata* to *porrecta*, with hyphae longitudinally arranged, loosely interwoven, septate, hyphae 3–6 µm in diam., hyphae becoming more compact toward the outside, colorless and hyaline. Toward the exterior, hyphae gradually widen and transitioning to *textura long-ellipsoid* to *textura prismatica*, or irregularly rounded cells to *textura subglobulosa*, with cells 12.5–35 × 5.5–11.5 µm. The outermost layer brownish incrusted.

##### Ecology.

On soil, among mosses, in a fir-dominated forest, scattered to gregarious; summer and fall.

##### Distribution.

China (southeastern Xizang and southwestern Gansu), mid-montane to subalpine.

##### Additional collections examined.

China • Gansu, Gannan Tibetan Autonomous Prefecture, Zhuoni County, alt. 2,927 m, in a mixed coniferous and broad-leaved forest, including spruce and birch trees, 2 September 2025, Jin-Ming Zhang, Zhjm1973 (HKAS 154397); • Xizang, Linzhi City, Gongbujiangda County, alt. 3,498 m, in a mixed forest dominated by *Picea asperata*, with gravel trees and rosaceous shrubs, on soil surrounded by mosses and humus, 9 August 2025, Jin-Rong Lu, LJR1573b (HKAS 135874).

##### Notes.

The phylogenetic species *Cudonia* sp. 1 (C281, H672, H664, C312, C286, C289, C290, and C291) was recognized by Ge et al. (2014) based on Asian and European specimens. Three newly collected specimens (HKAS 135873, HKAS 135874, and HKAS 154397) from Gansu and Xizang Provinces nested within *Cudonia* sp. 1 with strong support (SH-aLRT = 100, UFB = 100, BIPP = 1), suggesting that they are conspecific with this lineage despite the genetic distance among specimens. They represented an undescribed species and are formally named here as *C. pallida*. This species is closely related to *C. linzhiensis* (HKAS 133627 and HKAS 133645) and *C. lutea* (C280 and C283). Morphologically, the fruiting bodies of *C. pallida* were unique and distinguished from those of other known species of *Cudonia* by the warm, delicate, and smooth ascigerous portion. Compared to *C. pallida*, *C. linzhiensis* has a taupe to dark brown stipe when mature (Lu et al. 2026), and *C. lutea* has overall yellow fruiting bodies (Ge et al. 2014).

#### Cudonia subalpina

Taxon classificationFungiRhytismatalesCudoniaceae

J.R. Lu, F.M. Yu, J.F. Liang & Q. Zhao sp. nov.

FB941E02-A497-5DFD-8739-D8CE0EADCEF3

Index Fungorum: IF905591

Figs 4, 5

##### Chinese name.

亚高山地锤菌 (ya gao shan di chui jun).

**Figure 4.**
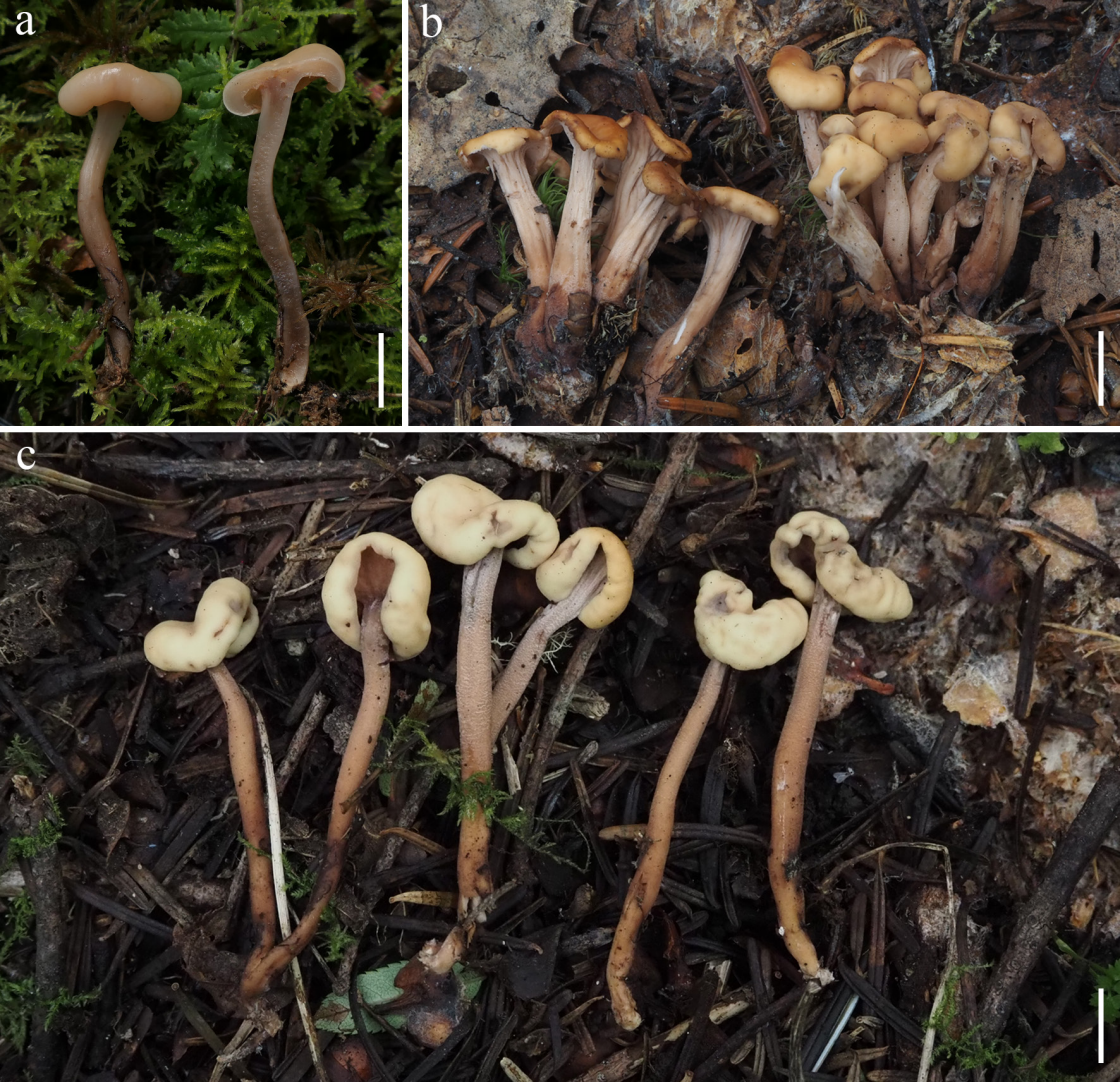
Fresh fruiting bodies of *Cudonia subalpina*. **a**. HKAS 135870; **b**. HKAS 135872; **c**. HKAS 135871 (holotype). Scale bars: 1 cm (**a–c**).

**Figure 5.**
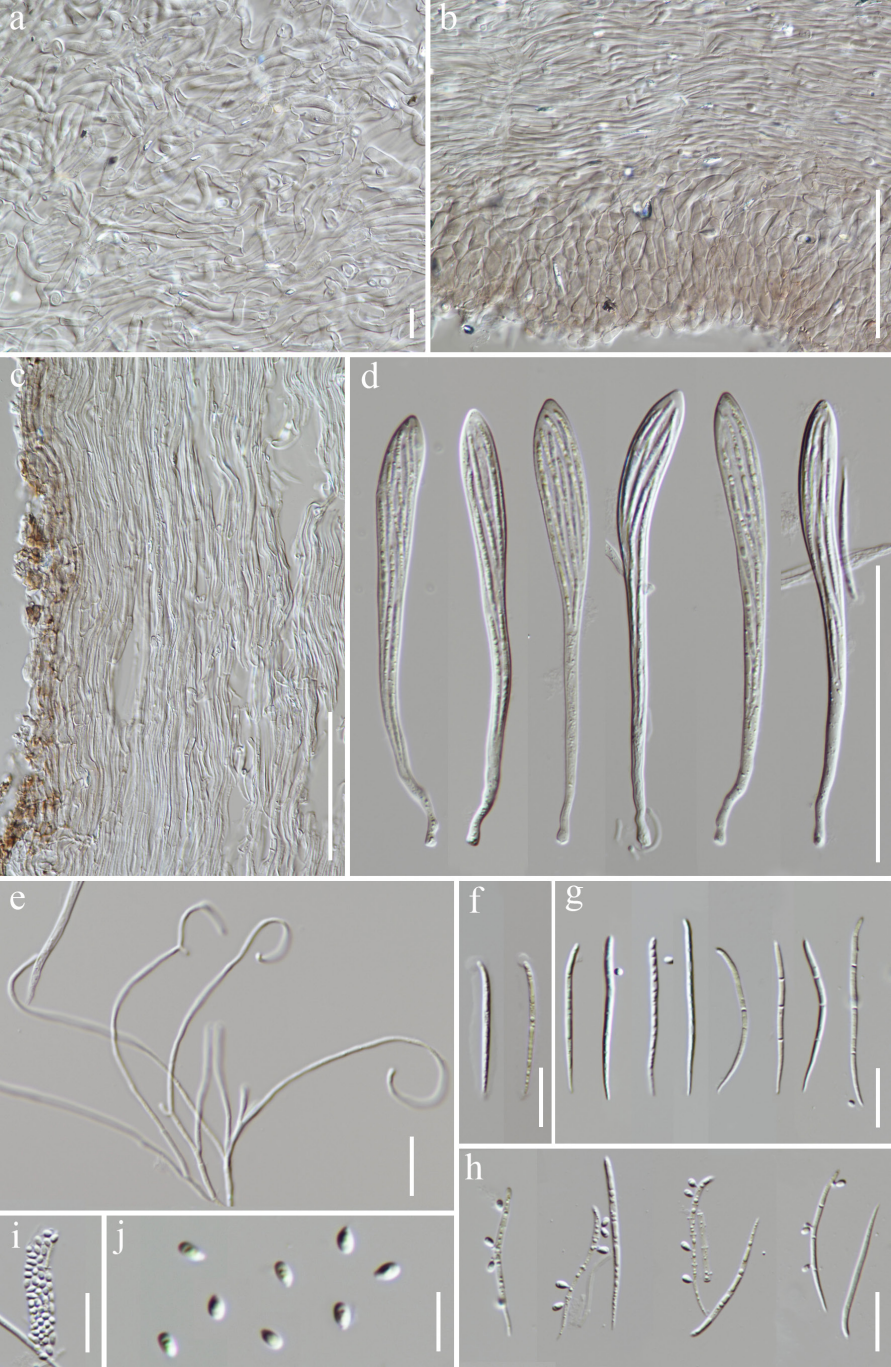
*Cudonia subalpina* [**a, b**. From HKAS 135870; **c**–**f**. From HKAS 135871 (holotype); **g**–**j**. From HKAS 135872]. **a**. Interior of the ascigerous portion; **b**. Outermost layer of the ascigerous portion (the side opposite the hymenium); **c**. Longitudinal section of stipe, showing the trama and surface (left side); **d**. Asci; **e**. Paraphyses; **f**. Ascospores with gelatinous sheaths and caps; **g**. Ascospores without gelatinous sheaths or caps; **h**. Ascospores producing ascoconidia; **i**. Portion of asci containing ascoconidia; **j**. Ascoconidia. Scale bars: 20 μm (**a, e–i**); 100 μm (**b–d**); 10 μm (**j**).

##### Etymology.

The specific epithet subalpina (Latin) refers to habitats of the fungus.

##### Holotype.

China • Xizang, Linzhi City, Bayi District, alt. 3,832 m, among mosses and humus under fir trees (dominated by *Abies fabri* and *Picea asperata*), 1 August 2024, Jin-Rong Lu, LJR 739 (HKAS 135871).

##### Diagnosis.

Similar to *Cudonia furfuracea* but differs in that it has darker ascocarps and stipes and wider ascospores.

##### Description.

***Ascocarp*** scattered to gregarious, capitate, stipitate, 3–6.5 cm in height. ***Ascigerous portion*** 1–2 cm in diam., capitate, margin entire or lobed to dentate; surface smooth when young, sometimes wrinkled, color dirty white, flesh-pink to orange-brown, texture moist and somewhat translucent; shape variable at maturity, irregularly compressed, often saddle-shaped, triangular or brain-like; color turning grayish-white to brownish-yellow, texture becoming firm and solid. ***Stipe*** 2.5–5.5 cm in height, 3–5 mm in diam., solid, erect or slightly bent, cylindrical to subcylindrical, brown to brownish-brown in the lower and middle parts, gradually transitioning upward to white to grayish-white; surface smooth when young, becoming distinctly longitudinally striate to ridged with age, the upper half often covered with white to brown furfuraceous (mealy) squamules. ***Stromatic membrane*** over the hymenium not observed.

***Hymenium*** 107.5–154.5 µm thick, consisting of paraphyses and asci at different stages of development. ***Asci*** [50/3/3] (109–)130–158 × 9–15(–17) µm, clavate, tapered towards the base, 8-spored, apical portion narrowly rounded, apical pore J^-^, croziers present. ***Ascospores*** [50/3/3] 39–57 × 2–3 µm, linear, clavate-filiform, acicular, often flexuous, rounded at apex and acuminate at base, multi-guttulate or none, 0–1–3–5-septate or more, hyaline and colorless; some ascospores with gelatinous caps at the apex, and covered with the gelatinous sheaths (2–4 µm thick in 5% KOH). ***Ascoconidia*** [50/3/3] 3–4.5 × 2–3 µm, obovoid, limoniform, smooth, 1-celled, containing a large oil drop, colorless and hyaline; sometimes nearly filling the asci. ***Paraphyses*** filiform, distantly septate, simple or branched below, upper part slightly to strongly curved, apical portion normal or slightly swollen; middle portion 1.5–2.5 µm diam., apical portion 2.5–3 µm diam. at the widest point, hyaline and colorless. ***Interior of ascigerous portion*** of *textura intricata*, loosely interwoven, hyphae 2.5–5 µm in diam., some swollen up to 8.5 µm in diam., colorless and hyaline. ***Outermost layer of ascigerous portion*** (the side opposite the hymenium) composed of gelatinized, subglobose, irregular rounded cells without sharp edges/angles, to ellipsoid, long-ellipsoid cells, 10.5–30 × 4.5–12.5 µm, arranged in short chains and perpendicular to the outer surface, covered a thin layer composed of smaller-sized subglobose, globose, ellipsoid cells, 5–9 × 4.5–7 µm or smaller, hyaline when isolated, becoming light brown and brownish when aggregated. ***Interior of stipe*** of *textura intricata* to *porrecta*, with longitudinally arranged, loosely interwoven hyphae becoming more compact toward the outside, septate, hyphae 3–4.5 µm in diam., colorless and hyaline. ***Surface of stipe*** covered with a discontinuous layer with short, curved, rectangular hyphoid cells, 1.5–3 µm in diam., mixed with subglobose, irregular rounded cells, to ellipsoid cells, 5.5–13 × 3.5–9 µm, the outermost cells brownish incrusted.

##### Ecology.

On soil covered with a thick humus layer, sometimes among mosses, in a fir-dominated forest, scattered to gregarious; summer.

##### Distribution.

China (southeastern and southern Xizang), subalpine.

##### Additional collections examined.

China • Xizang, Linzhi City, Bayi District, alt. 3,422 m, among mosses and humus under fir (dominated by *Picea asperata*) and gravel trees, 4 August 2025, Jin-Rong Lu, LJR 1471 (HKAS 135872); • Xizang, Shannan City, Luozha County, alt. 3,833 m, among mosses and humus under fir trees (dominated by *Picea asperata*), 31 July 2023, Jin-Rong Lu, LJR 493 (HKAS 135870).

##### Notes.

Phylogenetically, the species corresponds to “*Cudonia* sp. 3” in Ge et al. (2014), which indicates its independent phylogenetic position. It is closely related to *Cudonia circinans* (C316, C319, and C317), *Cudonia* sp. 11 (H340, H027, S9, and H037), and *Cudonia* sp. 7 (H670). *Cudonia circinans* has a cosmopolitan distribution, occurring most frequently in Europe and North America, whereas *Cudonia* sp. 11 is known only from North America and *Cudonia* sp. 7 is known only from China (northern temperate regions). This phylogenetic relationship suggests a strong temperate affinity for this subalpine species on the Qinghai-Xizang Plateau. Morphologically, *C. circinans* can be distinguished from *C. subalpina* by its dark brown stipe (Ge et al. 2014).

*Cudonia subalpina* is typically distributed in subalpine regions. Its specimens were found in coniferous forests at altitudes above 3,400 m. The morphological characteristics of this species vary from young to mature and then to aging stages, making it challenging to determine from its appearance alone whether all observed individuals belong to the same species. For example, the stipe is cylindrical to subcylindrical at the young stage, then becomes striate to ridged with age (especially in the upper part), and may eventually become entirely ridged in some cases. The gross morphology of *C. subalpina* is somewhat similar to that of *C. cangshanensis* (Niu et al. 2025). However, they are not closely related in the phylogenetic tree. Morphologically, *C. subalpina* differs by having longer asci on average (140.1 × 12.6 μm in *C. subalpina* vs. 120 × 12.3 μm in *C. cangshanensis*) and shorter ascospores (39–57 × 2–3 µm in *C. subalpina* vs. 52–82 × 2.1–4.3 μm in *C. cangshanensis*) (Niu et al. 2025).

The three new species proposed here are phylogenetically distant from one another (Fig. 1) and can be easily distinguished by their morphology. The anatomical structure of the ascigerous portion and the number of bud points on ascospores that produce ascoconidia may serve as useful characters for identifying *Cudonia* species. *Cudonia longispora* and *C. subalpina* share a similar anatomical structure of the outermost layer of the ascigerous portion, which differs from that of *C. pallida*. Among these three species, *C. longispora* has the longest ascospores and also exhibits the highest number of bud points producing ascoconidia on ascospores.

## Discussion

In the Qinghai-Xizang Plateau, recent investigations have documented substantial fungal diversity, encompassing both microfungi and macrofungi affiliated with the phyla *Ascomycota* and *Basidiomycota* (Yu et al. 2024; Wang et al. 2025; Xu et al. 2025; Lu et al. 2026). These findings have attracted significant research attention. Among these groups, the genus *Cudonia*, an old genus, has recently received renewed interest (Ge et al. 2014; Yang 2023; Niu et al. 2025; Lu et al. 2026).

Multilocus phylogenetic analyses combined with the Automatic Barcode Gap Discovery (ABGD) method revealed that species diversity in the genus *Cudonia* and its sister genus *Spathularia* is higher than previously recognized (Ge et al. 2014). Ge et al. (2014) delimited 23 phylogenetic species within this clade and identified East Asia as a major center of diversity. Subsequently, Yang (2023) described two phylogenetic species and formalized their nomenclature. Later, Lu et al. (2026) proposed a novel species, *C. linzhiensis*, based on two collections from Linzhi, Xizang. Compared with the sequence data, *C. linzhiensis* showed minor base differences from a previously reported *Cudonia* sp. 5, with only 5 bp (1.14%) differences in the ITS region and 1 bp (0.12%) difference in the LSU region (Lu et al. 2026). Only one specimen has been reported for *Cudonia* sp. 5 in the original research, collected from Yunnan, but it lacks morphological documentation. Given these few base differences, Lu et al. (2026) hypothesized that the two represent the same species. Genetic distances within *Cudonia* were calculated here using the Kimura 2-parameter model implemented in MEGA-X. The intraspecific ITS distance for *C. linzhiensis* (based solely on its new collections HKAS 133627 and HKAS 133645, Lu et al. 2026) is zero; when the *Cudonia* sp. 5 specimen (H658) is included, the distance increases to 0.0077 ± 0.0036. For the ITS region within the genus *Cudonia*, if *C. linzhiensis* and *Cudonia* sp. 5 are treated as two distinct lineages, the intragroup genetic distances range from 0 to 0.0049 ± 0.0029 (for *C. confusa*), and the intergroup genetic distances range from 0.0034 ± 0.0025 (between *C. circinans* and *Cudonia* sp. 11) to 0.089958 ± 0.016903 (between *C. gracilistipitata* and *Cudonia* sp. 14). The intergroup genetic distance between *C. linzhiensis* and *Cudonia* sp. 5 is 0.0116 ± 0.0052, which falls within the observed intergroup range. Under these circumstances, *C. linzhiensis* and *Cudonia* sp. 5 would be better represented as two different taxa. However, because the maximum intragroup genetic distance (0.0049 ± 0.0029) is greater than the minimum intergroup genetic distance (0.0034 ± 0.0025), there is overlap between intra- and intergroup genetic distances. Therefore, although the ITS region is a widely used standard DNA barcode in fungi, it is insufficient to distinguish all species within the genus *Cudonia*.

Beyond the limitations of the ITS barcode, issues of monophyly further challenge species delimitation in this genus. For example, Ge et al. (2014) treated two specimens collected from Yunnan as *Cudonia* sp. 6. However, as more collections became available, the putative phylogenetic species *Cudonia* sp. 6 was found not to be monophyletic. The present phylogenetic analyses revealed that *C. gracilistipitata*, *C. yunnanensis*, *Cudonia* sp. 6 (H680), and *Cudonia* sp. 6 (1222) formed a well-supported branch (Fig. 1). The genetic distance of the ITS region between H680 and 1222 is 0.0114 ± 0.0051, suggesting that these two specimens should belong to different species.

In this study, three newly collected specimens of *C. subalpina* were nested within *Cudonia* sp. 3. Sequence comparisons between HKAS 135871 and *Cudonia* sp. 3 (S11) revealed only 3 bp differences across 439 bp in the ITS region. The intragroup genetic distance of *C. subalpina* (including seven specimens of *Cudonia* sp. 3) is 0.0020 ± 0.0013, which is lower than the smallest observed intergroup genetic distances. This further supports the close affinity between our newly collected specimen and the previously documented *Cudonia* sp. 3, suggesting they represent the same taxonomic unit.

Descriptive habits for the microscopic structures of *Cudonia* species vary among researchers. For instance, when describing the structure of the ascigerous portion, some authors prefer using terms such as “medullary excipulum” and “ectal excipulum,” whereas others tend to directly describe the inner and outer morphology of the structure. Regardless of the terminology used, the outermost layer of the ascigerous portion deserves more emphasis in the genus *Cudonia*, as it can serve as a key feature for species identification, i.e., *C. pallida* bears a well-developed gelatinized layer in the outermost layer (as the ectal excipulum) of its ascigerous portion. Similarly, the number of bud points per ascospore for the production of ascoconidia has also received attention as a potential diagnostic character.

For some historical species, microstructural descriptions remain incomplete due to constraints imposed by contemporary technological limitations and resource availability. *Cudonia japonica*, for instance, lacks both ascoconidial descriptions and molecular data, thereby complicating comparative taxonomic studies and the delimitation of morphologically similar species. Additionally, although the family *Cudoniaceae* has been hypothesized to have originated in East Asia during the Paleogene (ca. 28 Mya), with subsequent radiation driven by Oligocene geological and climatic events (Ge et al. 2014), rigorous evaluation of this hypothesis necessitates comprehensive taxon sampling, including the integration of historical species such as *C. japonica* with modern collections. To address these deficiencies, future studies should prioritize expanded sampling from different geographic regions and ecological niches, particularly in the Qinghai-Xizang Plateau, one of the world’s biodiversity hotspots. The recollection of known species from type localities is essential for neotypification, whereas re-examination of historical herbarium specimens will facilitate the generation of supplementary morphological data and molecular sequences required for refined natural classification. Furthermore, more effective DNA barcodes are still needed to improve species-level resolution within this genus.

## Supplementary Material

XML Treatment for Cudonia longispora

XML Treatment for Cudonia pallida

XML Treatment for Cudonia subalpina

## Additional information

### Artificial Intelligence (AI) use

The authors accept full responsibility for the content of the manuscript, including the disclosure of any use of AI.

No AI tools were used in the preparation of this manuscript.

### Author ORCIDs

J.-R. Lu https://orcid.org/0009-0000-1094-3254

F.-M. Yu https://orcid.org/0000-0001-9133-8645

J.-M. Zhang https://orcid.org/0009-0002-6902-2585

Q. Zhao https://orcid.org/0000-0001-8169-0573

J.-F. Liang https://orcid.org/0000-0002-0433-2577

### Data availability

All of the data that support the findings of this study are available in the main text.
